# Parainguinal or Spigelian hernia: a clinically important distinction

**DOI:** 10.1007/s10029-025-03574-7

**Published:** 2026-02-17

**Authors:** Shanxuan Yu, Nazim Bhimani, Nicola Dodds, Edmund Sweeney, Simon Wickins, Anthony Glover, Thomas J Hugh

**Affiliations:** 1 Northern Clinical School, The University of Sydney, St Leonards, New South Wales Australia; 2 Upper Gastrointestinal Surgical Unit, Royal North Shore Hospital and North Shore Private Hospital, St Leonards, New South Wales Australia; 3 Faculty of Medicine and Health, The University of Sydney, Camperdown, New South Wales Australia; 4 The Tom Reeve Academic Surgical Clinic, Royal North Shore Hospital, 10 Westbourne Street, New South Wales 2065 St Leonards, Australia; 5 Department of Anaesthesia, North Shore Private Hospital, New South Wales St Leonards, Australia

**Keywords:** Hernia, Spigelian, Parainguinal, Herniorrhaphy, Surgical mesh, Postoperative complications

## Abstract

**Purpose:**

Parainguinal hernias (PHs) are an uncommon but under-recognised subtype of lateral abdominal wall hernia, located near the ASIS, distinct from the deep inguinal ring and at or just below the interspinous plane. They are frequently misclassified as Spigelian hernias (SHs) or inguinal hernias due to overlapping features. This study aimed to define the clinical characteristics, diagnostic accuracy, operative management, and long-term outcomes of patients with PHs compared with SHs.

**Methods:**

A retrospective cohort study of all adult patients undergoing mesh repair for PHs or SHs under the care of a single surgeon between 2002 and 2025 was undertaken. Clinical, radiological, and operative findings were analysed alongside complications and long-term outcomes. Pain outcomes were assessed using Cunningham’s criteria.

**Results:**

Forty-five patients underwent surgical repair: 40 with PHs and five with SHs (41 PH and 5 SH repairs). PH patients were older than SH patients (median 72 vs. 58 years, *p* = 0.036). PHs were more often left-sided (63%) and commonly misdiagnosed preoperatively as SHs (51%). Clinical diagnosis of PHs showed moderate sensitivity (56%) but high positive predictive value (96%), while CT and ultrasound performed poorly (sensitivities 6–15%). SHs were more reliably identified clinically (80%) but had low predictive values. PHs were repaired predominantly with open or laparoscopy-assisted mesh repair (95%), while SHs were repaired laparoscopically in most cases (80%). Complications were rare (seroma 4%, TIA 2%). At a median follow-up of 8.8 years (IQR 2.4–12.5), 87% of patients reported no pain, 13% had mild symptoms, and there were two recurrences (4%).

**Conclusion:**

PHs are more common than SHs, and recognition as a separate subtype is warranted to improve diagnostic accuracy and guide tailored management. In this series, open extraperitoneal mesh repair of PHs was associated with low complication rates, and excellent long-term patient outcomes.

## Introduction

Multiple groin hernia classification systems exist including the Gilbert, Nyhus, Schumpelick, Zollinger and the European Hernia Society (EHS) systems [1–5]. However, none have been universally adopted, partly reflecting anatomical complexity and variable presentations. The EHS system (2007) is widely used in research and registries, but a 2023 HerniaSurge update again highlights the absence of a globally accepted, simple, clinically applicable classification [1, 6]. In the EHS groin framework, lateral (indirect) hernias arise through the deep inguinal ring and run within the spermatic cord, lateral to the inferior epigastric vessels, whereas medial (direct) hernias protrude medial to these vessels through a weakness in the posterior wall of the inguinal canal [1]. Primary abdominal wall hernias are separately grouped as midline (umbilical, epigastric) or lateral (Spigelian, lumbar) [7].

**Fig. 1:**
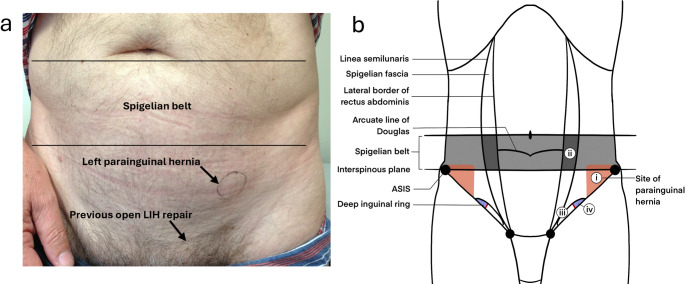
(**a**) Surface anatomy of a left PH and (**b**) schematic depicting the site of (i) a parainguinal hernia located inferior to the interspinous plane and Spigelian belt, medial to the ASIS, and superior and lateral to the inguinal canal, in comparison to the site of (ii) typical Spigelian hernias through the spigelian fascia, (iii) direct inguinal hernias within Hesselbach’s triangle and (iv) indirect inguinal hernias at the deep inguinal ring

True Spigelian hernias (SHs) occur through the Spigelian fascia along the semilunar line, typically within the “Spigelian belt” above the interspinous plane. The intraparietal course of these hernias beneath an intact external oblique and variability around the arcuate line contribute to diagnostic difficulty [8–12]. A subset of lateral abdominal wall hernias occurring at or below the inferior margin of the Spigelian belt, particularly near the Anterior Superior Iliac Spine (ASIS), are anatomically distinct from those arising within the classical Spigelian fascia. Historically, they have been labelled inconsistently (para-inguinal, peri-inguinal, “atypical” Spigelian or interstitial groin hernias), with reports spanning early descriptions by La Chausse, Holloway, Lower and Hicken, and Grierson and Leacock to later cohort series and reviews [9, 13–24]. These defects are situated adjacent to but separate from the inguinal canal, distinguishing them from both inguinal and Spigelian hernias (Fig. 1b). The term parainguinal hernia most accurately reflects this relationship, ‘para’ (from Greek, meaning beside or near to) denoting proximity without continuity. In contrast, the term ‘interstitial groin hernia,’ occasionally used in the literature, does not sufficiently describe this relationship, whilst the term ‘peri-inguinal’ misleadingly implies involvement of the inguinal canal itself [9].

We hypothesised that these parainguinal hernias (PHs), are more common than typical SHs and are frequently misdiagnosed due to limited awareness of this distinct entity. We therefore evaluated diagnostic pathways, operative findings and outcomes for patients undergoing surgical repair of PHs or SHs over 23 years in a single centre.

## Methods

A retrospective cohort study including patients ≥ 18 years of age who underwent repair of a parainguinal or Spigelian hernia under the care of a single surgeon between November 2002 and June 2025 was conducted. All operations were performed or supervised by the senior author. Data were analysed retrospectively from prospectively stored information. Ethics approval was obtained from the local Human Research Ethics Committee (2019/PID00066).

Surgeon correspondence and operation reports were reviewed to collect clinical data and operative details. Data collected and analysed included patient demographics, comorbidities, presenting symptoms, examination findings, imaging modality and interpretation as reported by radiologists, operative approach, hernia location and characteristics, mesh types, and postoperative outcomes. As data on smoking was not well documented, it was not included in the analysis. Due to the small number of PHs that were repaired laparoscopically, the efficacy of laparoscopic repair was not directly compared to open repair. Postoperative pain was assessed using Cunningham’s criteria and classified as either mild, moderate or severe [25]. At last follow up, patient reported outcome measures (PROMs) were assessed by a structured telephone interview or in clinic, including any recurrent swelling, ongoing groin pain classified using Cunningham’s criteria, or hernia recurrence.

Hernias were classified as either parainguinal or Spigelian based on intraoperative anatomical findings. Spigelian hernias were classified when located within the Spigelian belt and around the semi-circular line of Douglas (the arcuate line). For this study, a parainguinal hernia was defined as a lateral intraparietal defect located medial to the ASIS, lateral to the deep inguinal ring and within 1 cm above or below the inferior border of the Spigelian belt at the interspinous plane (Fig. 1; Table 4). These hernias are distinct from inguinal hernias, and from classical Spigelian hernias which usually arise through the Spigelian fascia well above the interspinous plane.

In this cohort, surgical site infection (SSI) was defined broadly to include the presence of any ‘erythema’ in or around the wound or by culture of an organism from a wound swab. Given the presence of mesh, all patients with a suspected SSI were treated early with oral antibiotics.

Categorical data were presented with a count and percentage and analysed using a Fisher’s Exact test. Continuous data were presented as a median and interquartile range and analysed using a Mann-Whitney U test. Statistical analysis was conducted on IBM Statistical Package for the Social Sciences version 29.0.1.0 (171) with statistical significance defined as *p* < 0.05. Demographics and comorbidities were recorded at the initial presentation while clinical, operative and outcome details were recorded for each hernia occurrence.

### Perioperative management

Patients who were smokers were counselled to stop before surgery. All patients received a single dose of intravenous ceftriaxone and flucloxacillin at induction to minimise infection, and patients with penicillin allergy were given alternatives. Alcohol-based skin preparation was used in all patients.

### Operative approach and technique

**Fig. 2:**
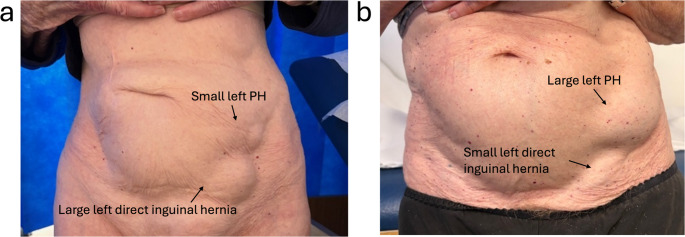
Surface anatomy of left PHs with coexistent inguinal hernias, with PH hernia orifices originating lateral to and separate from the deep inguinal ring on dissection

**Fig. 3:**
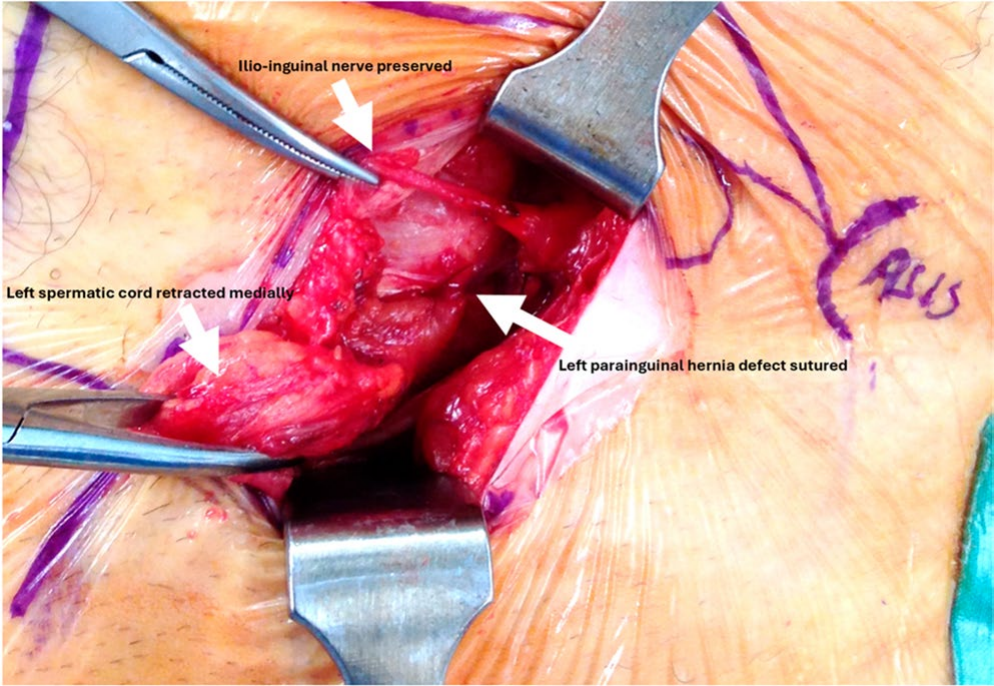
Left PH with the preserved ilio-inguinal nerve, and the hernia defect sutured closed lying lateral to and separate from the spermatic cord

**Fig. 4:**
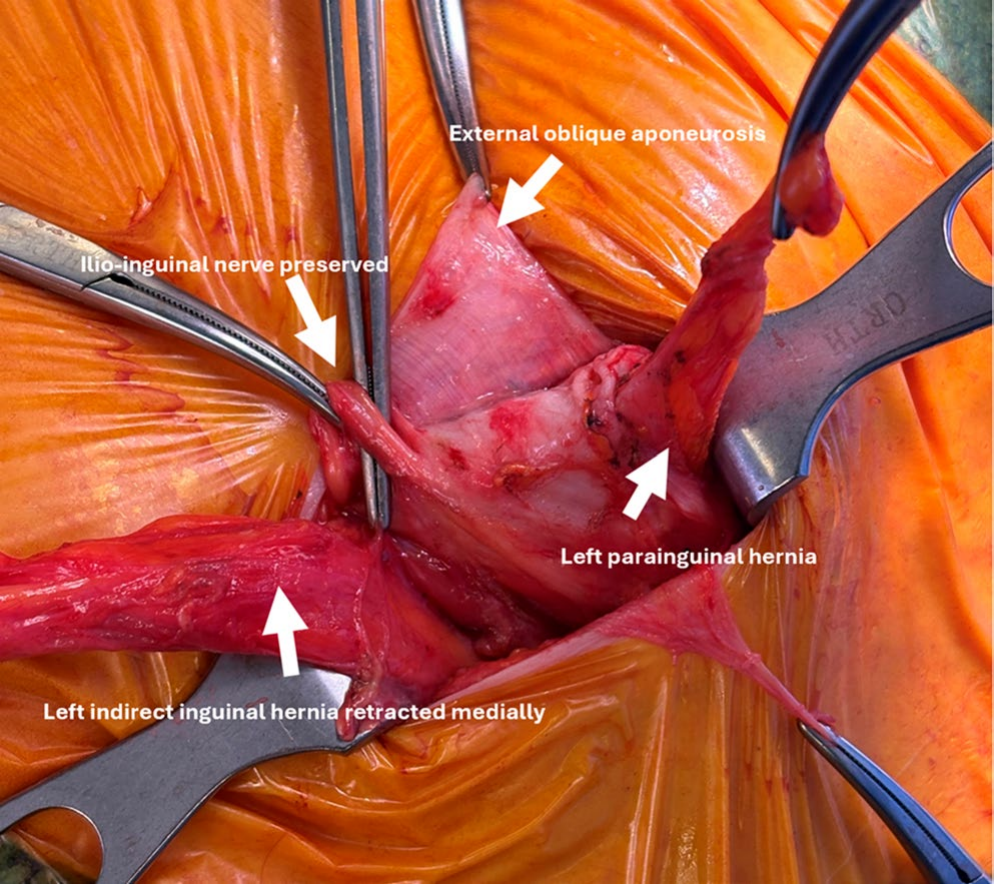
Left PH containing fat as seen at open repair with the ilio-inguinal nerve preserved

**Fig. 5:**
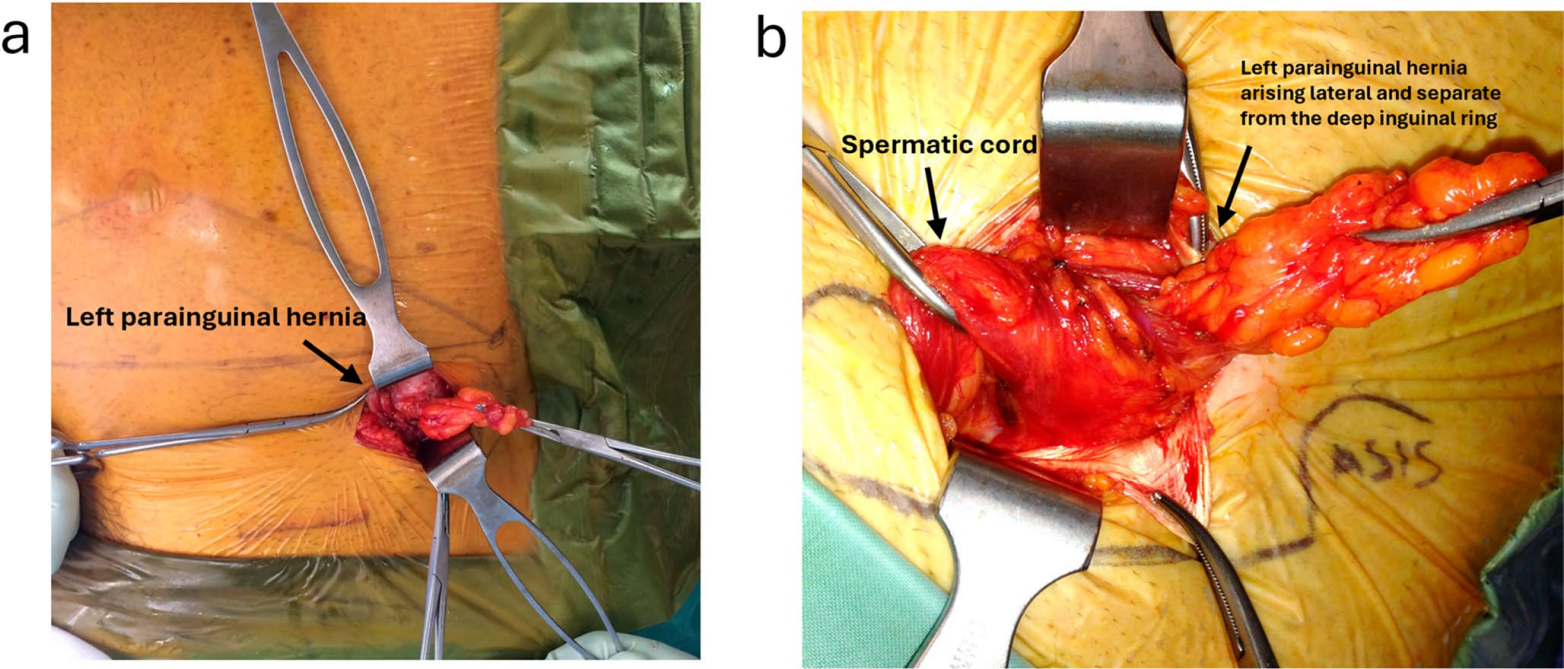
Mobilisation of two different left PHs during a day-only open mesh repair

For clinically or radiologically obvious PHs (Figs. 1 and 2), an open mesh repair was performed under either local anaesthesia with sedation or general anaesthesia. Standard operative steps included: (a) pre-emptive skin and subcutaneous infiltration with 4–5 mLs of 0.25% Ropivacaine (Astra-Zeneca, Cambridge, UK); (b) 3–4 cm transverse incision over the hernia; (c) diathermy dissection through Scarpa’s fascia to expose the external oblique (EO) aponeurosis; (d) infiltration of 2–3 mLs of 0.25% Ropivacaine (Astra-Zeneca, Cambridge, UK) beneath the EO aponeurosis; (e) knife dissection of the EO to identify and preserve any sensory nerves (Figs. 3 and 4); (f) identification, mobilisation, and either reduction or excision of the hernia sac (Figs. 4 and 5); (g) suture closure of the hernia defect with an 0 PDS suture (Fig. 3); (h) placement of a polyester ProGrip™ mesh over the defect fixed with several 2/0 Prolene sutures; (i) placement of a fine-bore catheter into the space to provide an infusion of 0.25% Ropivacaine (Astra-Zeneca, Cambridge, UK) for 48 h postoperatively; (j) closure of the EO and Scarpa’s fascia with a 2/0 vicryl suture; (k) subcuticular skin closure with a 4/0 vicryl suture. For patients with a clinically or radiologically obvious Spigelian hernia, a laparoscopic intraperitoneal onlay mesh (IPOM) repair was preferred using a technique described previously [26].

**Fig. 6:**
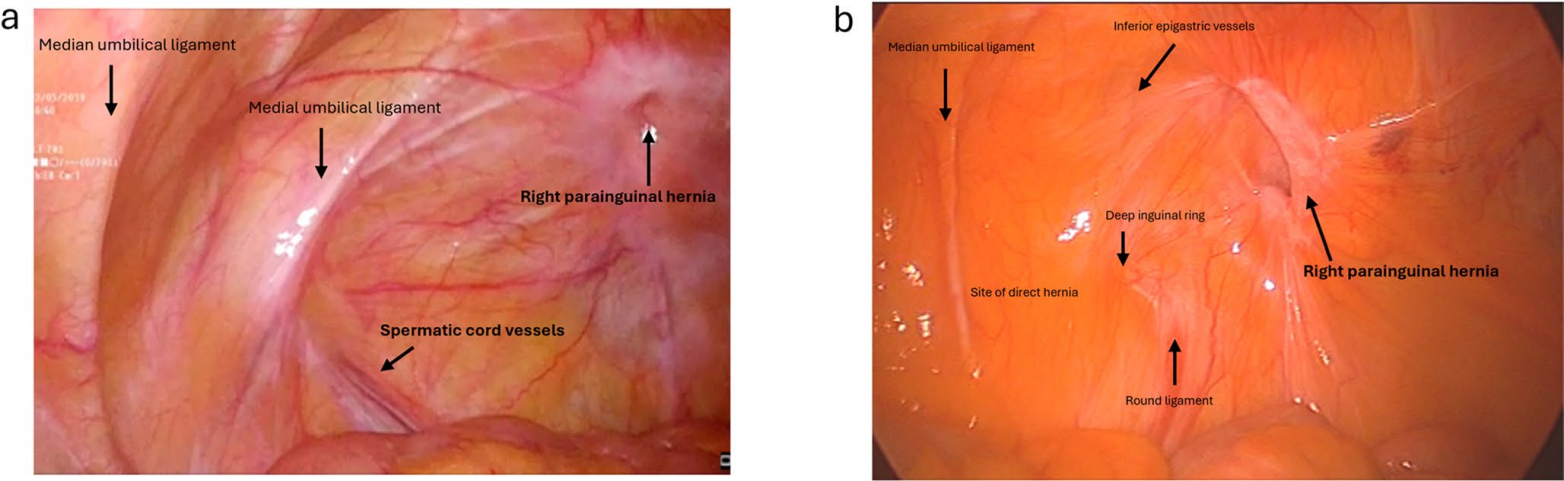
Laparoscopic view of right PHs with the hernia defect lying lateral to the deep inguinal ring in a (**a**) male patient and (**b**) female patient

Where there was diagnostic uncertainty, a laparoscopy was performed to enable direct inspection of the anterior abdominal wall to discern whether a PH or SH was present (Fig. 6). If a true SH was identified, the procedure continued laparoscopically as an IPOM repair, whereas if a true PH was identified, the diagnostic laparoscopy was concluded and a small open anterior extraperitoneal mesh repaired followed using the technique described above. This approach was selected because the parainguinal defect lay immediately medial to the ASIS, and although a laparoscopic approach is possible, it may be more technically challenging and increases the risk of injury to the lateral cutaneous nerve of the thigh compared to an anterior approach. A small anterior incision allowed direct repair of the defect and mesh placement between the internal oblique and the external oblique aponeuroses without breaching the peritoneal cavity.

Detailed post-operative information brochures were provided prior to discharge, with specific self-care instructions about removing the analgesic catheter at home on the second postoperative day. Discharge criteria included the ability to mobilise freely, the ability to void, and being pain free. An analgesic pack containing paracetamol, a nonsteroidal anti-inflammatory agent and a stronger non-constipating oral medication was provided on discharge with a recommendation to take regular pain relief for 4–5 days. Patients were encouraged to return to normal activities as soon as possible and were reviewed in the clinic four weeks postoperatively and thereafter for any ongoing pain, or wound complications.

### Follow up

Routine post-operative clinical review was done at 4–6 weeks, and long-term follow-up was done by telephone interview. At long-term follow up, a semi-structured telephone interview focused on two patient-reported outcomes: the presence of a bulge, and the presence of pain or discomfort at the hernia site. Any further abdominal surgery at the hernia repair site was also noted. Patients who reported symptoms were reviewed in the clinic to exclude a recurrent hernia and to assess other PROMs, namely pain outcomes as defined by Cunningham’s criteria and other assessments [25], however no formal PROMs survey was used. Hernia recurrence was assessed based on observations from direct clinical examination, intra-operative findings during repeat operations, or by radiological confirmation.

## Results

### Patient demographics and operative findings

During the study period, 79 patients were referred with lateral lower abdominal swelling or pain and a suspected abdominal wall hernia. Fourteen were excluded because no hernia was identified at clinical examination (*n* = 7) or they elected to have non-operative management (*n* = 7). A further 20 were excluded after surgical intervention confirmed alternative diagnoses, including inguinal, femoral, or incisional hernias, or non-hernia pathology. The remaining 45 patients are the subject of this study: 40 with parainguinal hernias (PHs) and five with Spigelian hernias (SHs). One patient had bilateral PHs, making a total of 41 PHs.

The demographic, clinical, and operative characteristics are outlined in Table 1 and Appendix Tables 5 and 6. The median age at presentation was 71 years (IQR 65–78; range 42–89). Patients with PHs were older than those with SHs (72 vs. 58 years, *p* = 0.036). Sex distribution was equal in the parainguinal hernia (PH) group (20 male, 20 female), while SHs showed a male predominance (4:1). Just over half of the cohort (56%) had undergone prior abdominal surgery, and 40% reported a history of a previous hernia. Gastrointestinal (36%) and cardiovascular (31%) comorbidities were most common, but there were no significant associations between comorbidity profile and hernia type that could be identified from our cohorts (Appendix Table 5).

**Table 1:** Patient demographics and comorbidities at first presentation (*n* = 45)

	All	Parainguinal	Spigelian	*P*-value
Number	45	40	5	
Median Age/Years (IQR)	71 (65–78)	72 (66–78)	58 (53–71)	0.036
**Sex**				0.352
Male	24 (53.3%)	20 (50.0%)	4 (80.0%)	
Female	21 (46.7%)	20 (50.0%)	1 (20.0%)	
**Associated Factors**				
Past Abdominal Operations	25 (55.6%)	23 (57.5%)	2 (40.0%)	0.642
Previous Hernia	18 (40.0%)	16 (40.0%)	2 (40.0%)	0.999
**Comorbidities**				
Gastrointestinal	16 (35.6%)	16 (40.0%)	0	0.144
Cardiovascular	14 (31.1%)	13 (32.5%)	1 (20.0%)	0.999

Most hernias were left-sided (65%) and were palpable on examination (87%). PHs were most frequently referred as suspected Spigelian hernias (51%), while SHs were commonly referred for a lower abdominal swelling and a possible Spigelian hernia (SH) (60%). The predominant presenting features were swelling or asymmetry (85%) and pain or discomfort (59%) (Appendix Table 6).

Coexistent hernias were identified in 19 patients (41%), most often inguinal (*n* = 17) or periumbilical (*n* = 5). Among PH patients, 23 (56%) were correctly diagnosed pre-operatively based on clinical examination, while 17 (44%) were misclassified as Spigelian (34%), inguinal (7%), or incisional (2%). In contrast, four of five SHs (80%) were accurately diagnosed pre-operatively based on a clinical examination, while one was initially considered a PH. A comparison of the clinical and radiological diagnostic accuracy is summarised in Appendix Table 7. For PHs, clinical diagnosis showed moderate sensitivity (56%) and good specificity (80%), with a very high PPV (96%) but low NPV (18%). In comparison, CT and ultrasound performed poorly (sensitivities 6% and 15% respectively; NPVs 17% and 6%). For SHs, clinical diagnosis achieved high sensitivity (80%) and moderate specificity (66%), but PPV was low (22%) despite a high NPV (96%). CT and ultrasound again performed poorly, with low specificities (19% and 40%) and PPVs (19% and 8%) (Appendix Table 7).

### Operative and post-operative findings

The operative details of the cohort are shown in Appendix Table 6. The median operating time was slightly longer for SHs (55 min, IQR 43–79) compared to PHs (50 min, IQR 40–61), although this was not significant. PHs were typically managed with open mesh repair (*n* = 20, 49%) or combined diagnostic laparoscopy and open mesh repair (*n* = 19, 46%). Only two patients in this group had a laparoscopic IPOM repair (5%). In contrast, SHs were usually repaired as a laparoscopic IPOM (*n* = 4, 80%), with only one patient undergoing an open mesh repair (20%).

Most patients with PHs (*n* = 40/41, 98%) and SHs (*n* = 4/5, 80%) had their operations under general anaesthesia. Polyester ProGrip™ inlay mesh was used in most PH repairs (*n* = 37, 90%), while SH IPOM repairs used Gortex dual mesh (*n* = 4, 80%). Four patients (three PHs, one SH) presented with previously missed hernias after prior surgical interventions.

Postoperative complications were infrequent, with only two seromas (4%) and one transient ischaemic attack (TIA) (2%). Most patients (94%) had no complications, and most were discharged within 24 h of their operation (33% same day, 50% overnight, 17% >1 night; Table 2).

**Table 2:** Postoperative outcomes and complications (*n* = 46)

Details	All	Parainguinal	Spigelian	*P*-Value
**Complications **(*n* = 46)				0.999
Seroma	2 (4.3%)	2 (4.9%)	0	
TIA	1 (2.2%)	1 (2.4%)	0	
None	43 (93.5%)	38 (92.7%)	5 (100.0%)	
**Length of Stay** (*n* = 36)				
Same Day Discharge	12 (33.3%)	12 (34.3%)	0	
Overnight Stay	18 (50.0%)	17 (48.6%)	1 (100%)	
> 1 Night Stay	6 (16.7%)	6 (17.1%)	0	

### Follow up

At long-term follow-up (median 8.8 years, IQR 2.4–12.5; range 0.1–20.1), 87% of patients reported no pain or recurrent swelling, and with only 13% describing intermittent mild discomfort. No patients reported moderate or severe pain. During the follow up period, two patients developed recurrence (4%, one in each hernia group) (Table 3), both of whom were known to be non-smokers. Overall, both PH and SH repair was associated with durable long-term outcomes, with low complication and recurrence rates.

**Table 3:** Long-term follow-up outcomes (*n* = 46). Median follow-up period was 8.8 years (IQR 2.4–12.5; range 0.1–20.1)

Details	All	Parainguinal	Spigelian
Pain (Cunningham)^a^			
0 (none)	40 (87.0%)	35 (85.4%)	5 (100.0%)
1 (mild)	6 (13.0%)	6 (14.6%)	0
Recurrence	2 (4.3%)	1 (2.4%)	1 (20.0%)

^a^ 0 = None, 1 = mild without limitations to activity, 2 = moderate pain preventing return to preoperative lifestyle and 3 = severe pain incapacitating the patient at frequent intervals or interfering with activities of daily living

## Discussion

This study outlines the presentation, operative findings and long-term outcomes of patients with primary lateral abdominal wall hernias located near the ASIS. With a median follow up of 8.8 years, postoperative complications were rare, chronic pain was minimal, and there were only two recurrences (4.3%). In patients with PHs, open mesh repair through a small incision is a safe and effective treatment for this distinct group of lateral abdominal wall hernias. Although the small number of SHs in this retrospective study limits statistical comparison of outcomes between PHs and SHs, a key finding was that PHs predominated over true SHs and were often misclassified pre-operatively. Szasz et al. (2023) described similar low lateral abdominal wall defects as ‘interstitial groin hernias,’ located near the ASIS [9]. Based on operative correlation, we believe these correspond to what we classify as ‘parainguinal hernias’, distinct from the inguinal canal and, in our opinion better described by the prefix ‘para,’ indicating adjacency without involvement of the inguinal canal.

### Terminology and historical context

Reports of these hernias have referred to them using varying terminology, including ‘para-inguinal’, ‘peri-inguinal’, and ‘atypical’ inguinal or Spigelian variants, emphasising ongoing confusion in nomenclature and the need for clarification and a unified definition [16–18, 20–24]. In 1991, Gallese distinguished ‘peri-inguinal’ hernias from ‘para-inguinal’ hernias based on anatomical relationships to the inguinal canal, noting the consistent misclassification of para-inguinal hernias as atypical inguinal or Spigelian hernias due to their unusual location [19]. We used the term ‘parainguinal’ to describe these hernias, as the Greek word ‘para’ (beside, near to, distinct from) best reflects their consistent anatomical position, as opposed to ‘peri’ (around, about, enclosing). They arise adjacent to but separate from the inguinal canal and ASIS, distinct from the traditional site of Spigelian hernias which are usually situated higher in the abdominal wall (Figs. 1, 2, 3, 4 and 5; Table 4). Although a 1904 case report has been cited as the earliest modern use of this term, its origins trace back to 1746 when La Chausse described this distinct ventral hernia [13, 14].

**Table 4:** Comparison of classical Spigelian, inguinal, and parainguinal hernias

Feature	Classical Spigelian Hernia (SH)	Inguinal Hernia (IH)	Parainguinal Hernia (PH)
Anatomical location	Along the *Spigelian fascia* between the semilunar line and the lateral edge of the rectus sheath, usually above the interspinous plane (“Spigelian hernia belt”)	Arises within the inguinal canal, either lateral (indirect) or medial (direct) to the inferior epigastric vessels	Near or just inferior to the ASIS, lateral and *distinct from the deep inguinal ring*; typically below the interspinous plane
Relation to inferior epigastric vessels	Variable, but generally superior and lateral	Indirect: lateral Direct: medial	Lateral and separate from the deep ring; may lie adjacent to vessels but not through them
Relation to the inguinal canal	Separate from the canal; does not involve the spermatic cord	Hernia sac traverses or abuts the canal (via deep ring)	Lateral and adjacent to but distinct from the inguinal canal; external oblique often intact
Fascial defect site	Through the *Spigelian fascia*	Through the *transversalis fascia*	Through the *lateral intraparietal defect*

### Diagnosis and anatomy

Pre-operative diagnosis of PHs was challenging. Clinical assessment showed only moderate sensitivity but high positive predictive value, whereas CT and ultrasound performed poorly, consistent with recognised difficulty in detecting intraparietal hernias deep to an intact external oblique, particularly in obese patients [10–12, 24]. Dynamic imaging modalities such as real-time ultrasound or valsalva-enhanced CT may enhance preoperative detection of these intraparietal defects and warrants evaluation in future studies. Several patients in our study underwent negative explorations or prior hernia surgery before a PH was identified, highlighting potential delays and occasionally misdirection in care. In our patients, diagnostic laparoscopy was a helpful adjunct when clinical or radiological findings were equivocal [9, 12]. Intraoperative findings (open and laparoscopic) in this study consistently demonstrated that parainguinal hernia (PH) defects were located medial to the ASIS, typically within approximately 1 cm above or below the interspinous plane. These hernias were distinct from the deep inguinal ring and lay either at the inferior edge or below the Spigelian belt. While we consider PHs to be closely related to SHs, we regard them as a separate and clinically relevant variant rather than a subtype. This classification reflects consistent operative findings that distinguish PHs from both classical Spigelian and inguinal hernias (Fig. 1b; Table 4). Occasional coexistent indirect inguinal hernias were anatomically distinct (Figs. 2 and 4).

Although termed by some as atypical or variants of Spigelian hernias, parainguinal hernias as described in our patient cohort often lay beyond the Spigelian fascia, implying a potentially different pathophysiological origin [18]. Although the precise mechanism underlying PH development is uncertain, a plausible explanation may involve focal weakness at the interface between the Spigelian fascia and the attenuated internal oblique muscle at this level. This is a theoretical observation, and other mechanisms, including variations in the neurovascular penetration cannot be excluded. Historically, comparable low-lateral hernias have been reported under varied labels (para-inguinal, peri-inguinal, interparietal or atypical Spigelian) in both early literature and modern case series and reviews, highlighting persistent classification ambiguity and reinforcing the need for a unified concept [9, 13–24, 27–31].

### Operative implications

The main contribution of this study is not in technical innovation but in clarifying anatomical distinctions and encouraging refinement of existing hernia classification systems. Our contention is that lateral abdominal wall hernias occurring at or just below the inferior margin of the Spigelian belt, typically within 1 cm above or below the ASIS represent a consistent and clinically distinct variant of Spigelian hernia. While some authors describe the Spigelian fascia as extending to the pubic bone, the inferior portion is often attenuated or blended with the internal oblique aponeurosis, creating a potential weak point at this transition. We have therefore used the term parainguinal hernia to denote these defects, reflecting their proximity to the inguinal canal yet anatomical separation from it. This terminology, while not implying a different pathogenesis, highlights the unique operative and diagnostic characteristics of hernias occurring in this low lateral location. The additional schematic diagram clarifies this anatomical relationship and differentiates parainguinal hernias from classical Spigelian and inguinal hernias (Fig. 1b).

However, recognition of PHs also has practical implications. While laparoscopic repair is recommended for many hernias, our experience suggests that parainguinal hernias present distinct anatomical challenges. These defects are located immediately adjacent to the ASIS, near the lateral cutaneous nerve of the thigh and are anatomically remote from the inguinal canal. Consequently, a limited anterior extraperitoneal approach allows direct closure and mesh reinforcement with minimal dissection and negligible risk to sensory nerves. In contrast, laparoscopic repair in this location may require wide peritoneal mobilisation and fixation near neurovascular structures, potentially offsetting any theoretical advantages. Therefore, while a minimally invasive approach may be appropriate for selected patients in expert hands, we consider the anterior approach a safe and pragmatic option for most patients and our long-term results support this conclusion. By contrast, true SHs, although rarer and situated higher along the semilunar line, are well suited to minimally invasive repair using standard IPOM, TAPP or TEP techniques [32, 33].

In patients where there was diagnostic uncertainty preoperatively, diagnostic laparoscopy was valuable to confirm the defect location before proceeding to an open repair (Fig. 6).

## Conclusion

Despite extensive hernia literature, PHs are absent from contemporary classifications, perpetuating diagnostic uncertainty and likely inflating reports of SHs. While the single-surgeon retrospective design of our study ensures consistency of diagnosis and technique, it may limit wider applicability. Furthermore, sample sizes in this study were imbalanced, with the PH group being substantially larger than the SH group, and so conclusions about differences between the groups are limited. Multicentre prospective studies with imaging and operative correlation are warranted to refine anatomical boundaries and validate these findings, which may explore statistical comparisons in matched cohorts.

Our long-term experience supports the view that low lateral abdominal wall hernias occurring adjacent to the ASIS represent a reproducible and clinically significant subgroup within the Spigelian spectrum. These hernias have distinctive anatomical and operative features that justify clearer recognition and a defined position within future hernia classification systems. While not a newly discovered entity, parainguinal hernias have been historically under-reported and inconsistently labelled. Their formal recognition as a distinct category may improve diagnostic precision, facilitate appropriate operative planning, and enhance consistency in surgical reporting and registry data. Our results demonstrate that tailored small-incision extraperitoneal mesh repair provides safe and durable long-term outcomes for these patients.

## Appendix

**Table 5:** Clinical findings and reasons for referral (*n* = 46)

	All	Parainguinal	Spigelian	*P*-value
**Side**				0.645
Left	30 (65.2%)	26 (63.4%)	4 (80.0%)	
Right	16 (34.8%)	15 (36.6%)	1 (20.0%)	
**Reason for Referral**				0.327
Spigelian Hernia	22 (47.8%)	21 (51.2%)	1 (20.0%)	
Inguinal/Groin Hernia	11 (23.9%)	10 (24.4%)	1 (20.0%)	
Lower Abdominal Swelling/Hernia	10 (21.7%)	7 (17.1%)	3 (60.0%)	
Unusual Lower Abdominal Wall Hernia	2 (4.3%)	2 (4.9%)	0	
Incidental Diagnosis	1 (2.2%)	1 (2.4%)	0	
**Clinical/Examination Findings**				
Palpable Hernia/Defect	40 (87.0%)	37 (90.2%)	3 (60.0%)	0.120
Swelling/Asymmetry	39 (84.8%)	35 (85.4%)	4 (80.0%)	0.999
Pain/Discomfort	27 (58.7%)	24 (58.5%)	3 (60.0%)	0.999
Associated Hernia	19 (41.3%)	18 (43.9%)	1 (20.0%)	0.387

**Table 6:** Intraoperative details for parainguinal and Spigelian hernias

Intraoperative Details	Parainguinal	Spigelian	*P*-Value
Median Operating Time/mins (IQR)	50 (40–61)	55 (43–79)	0.511
**Operation Type**			< 0.001
Laparoscopy	2 (4.9%)	4 (80.0%)	
Open	20 (48.8%)	1 (20.0%)	
Laparoscopy & Open	19 (46.3%)	0	
**Anaesthetic**			0.017
General	40 (97.6%)	4 (80.0%)	
Local Only	1 (2.4%)	1 (20.0%)	
**Mesh Type**			< 0.001
ProGrip™	37 (90.2%)	0	
Gortex Dual Mesh	1 (2.4%)	4 (80.0%)	
Sandwich	3 (7.3%)	1 (20.0%)	
**Hernia Contents**			
Bowel	4 (9.8%)	0	0.999
Colon	7 (17.1%)	2 (40.0%)	0.248
Fat	14 (34.1%)	0	0.303
Omentum	4 (9.8%)	0	0.999
Cord Lipoma	1 (2.4%)	0	0.999
No Contents	12 (29.3%)	3 (60.0%)	0.633
Missed Hernia Requiring Reoperation	2 (4.9%)	1 (20.0%)	0.298

**Table 7:** Sensitivity/Specificity analysis of clinical examination (*n* = 46), CT (*n* = 21) and ultrasound (*n* = 20) for parainguinal and Spigelian hernias. Imaging results were documented as reported by radiologists

Hernia Type	Parainguinal			Spigelian		
Diagnosis Method	Clinical	CT	US	Clinical	CT	US
Sensitivity (%)	56.1	6.3	15.0	80.0	100.0*	100.0*
Specificity (%)	80.0	100.0*	100.0*	65.9	18.8	40.0
PPV (%)	95.8	100.0*	100.0*	22.2	18.8	7.7
NPV (%)	18.2	16.7	5.6	96.4	100.0*	100.0*

*Small denominators (*n* < 5)
